# Outlines of the Ancient History of Medicine, Being a View of the Healing Art among the Egyptians, Greeks, Romans, and Arabians

**Published:** 1831

**Authors:** 


I. Outlines of the Ancient History of Medicine, being a View of the Healing Art among the Egyptians, Greeks, Romans, and Arabians. By *D. M. Moir*, Surgeon. Blackwood, Edinburgh; and Cadel, London. 1831.

II. The History of Medicine, Surgery, and Anatomy, from the Creation of the World to the Commencement of the Nineteenth Century. By *W. Hamilton*, M.B. Two Volumes. London, Colburn and Bentley. 1831.

Before the appearance of Hippocrates medicine could not be dignified with the name, as it did not wear the character of science. Wrapped up in mythological mystery, and entangled within the mazy labyrinths of superstition, the facts to which it could pretend were few, and many of those were based upon no very settled principles. The gods being regarded as the first physicians, divination was liberally intermingled with all medical inquiries, and an act, which not only traced its origin to Heaven, but claimed the treatment of diseases, which were themselves regarded as of celestial birth, gave but little encouragement to the exercise of human industry. Apollo has been honoured by many with the fatherhood of physic, his reputed son Orpheus has been invested with the same distinction by others, the Centaur Chiron has put forward strong claim to the same merit, but Æsculapius has been esteemed by very general consent its most legitimate parent. According to Cicero these individuals, all of whom were distinguished by their medical attainments, were called by this name, while the veritable Æsculapius Galen claims for Greece; but, with the exception of a few scattered notices through the pages of ancient history, no record exists of the services which attained for him this high pre-eminence. On his demise, however, he was elevated by his cotemporaries to the mansion of the gods, his sons Podalirius and Machaon followed Achilles to the Trojan war, and his descendants, the Asclepiades, made themselves remarkable, for many centuries afterwards, for the care with which they recorded on the walls and pillars of their temples the cures which they performed, and the medicines by which these cures had been achieved. Had not priestcraft interfered with these primitive hospitals and rude journals of clinical medicine, by converting to its own aggrandisement the wealth and reputation which they were fast acquiring, medicine had certainly made more rapid progress; but finding that to combine the mysteries of their absurd idolatry with the promising allurements of the healing art, they acquired a stronger power over the human mind, they gradually appropriated to themselves the two professions, ignorantly blended them into one senseless mixture, and although more than five hundred years had elapsed between the siege of Troy and the birth of the Sage of Cos, it is truly melancholy to witness the unimproved and stationary condition of medicine during that lengthened period. With the exception of about half a dozen names, and the greater number of these were names without a name—history passess in silence over this unproductive era, and when we read some of the doctrines of Alcmæon, whom Aristotle, Diogenes, and Plutarch unite in lauding as the brightest luminary to which that age gave birth, it cannot be considered strange that the moderns should stop at Hippocrates in their retrospect of medical history. He is said to have been the first who engaged in dissection, but as the human body was then and for a long time after regarded sacred after death, his knife, there is good reason to believe, was exclusively confined to the inspection of inferior animals. Of chemistry nothing was known, unless we consider as knowledge the visionary efforts of the alchemist to transmute the metals into gold. The catalogue of medicines, which were commonly employed, was extremely meagre. The school of Cnidos almost confined itself to the use of elaterium and one or two other drastic purgatives, and for many of the most active remedies which they employed, they acknowledged themselves indebted to the favor of the gods. Thus Diana is supposed to have discovered the properties of wormwood, Circe the effects of nightshade, and Pallas the virtue of feverfew. The surgical knowledge of the ancients, which for obvious reasons must have been more complete than that of any other branch of medicine, seems to have principally consisted of phlebotomy, stopping hæmorrhage by styptics or cautery, binding up wounds and fractures, and removing by free incisions darts and arrows from the flesh. But the treatment of internal disease was so utterly unknown that, although Homer, speaking of Æsculapius, says that he was able to restore even the dead to life, because he succeeded in stanching the wounds of Hippolytus, is more than once compelled to invoke the assistance of the gods, to expel from the Grecian camp the ravages of pestilence.

In some such state as this was medicine when the beginning of the 80th Olympiad blessed the world with one of the brightest ornaments in the person of Hippocrates. This truly great man was the 17th in descent from Æsculapius, if the genealogies of the Asclepiadæ can be confided in, and as both his father and grandfather were physicians, he entered upon the study of his profession with advantages which his bold and original mind was well calculated to improve. The school of Cos, in which he received the rudiments of his education, was conducted on more philosophical principles than either that of Rhodes or Cnidos, which were its most formidable cotemporaries. Disease was more patiently studied in connexion with its proximate cause, and medicine was more carefully administered with relation to its effects upon the body. Priestcraft was exercised, however, in all the schools of the Asclepiadæ, and medicine was so wrapped up in the mysteries of divination, that senseless incantations, unmeaning prayers, and ostentatious ceremonies were as generally employed and as much confided in, as the skill of the physician or the power of drugs. The imagination could often be excited when the body was insensible to stimuli, and the comforts and consolations of the priest were not unfrequently more soothing to the patient than operations clumsily performed and medicines exhibited in ignorance. The sagacious mind of Hippocrates soon discovered the injurious influence of priestly interference, and one of the first principles, which he labored to establish, was, that diseases were not rained on us from Heaven, but were earth-born evils, and that therefore while prayer and penancies were excellent in themselves, they could exert but little power in their removal, while they tended to cramp human industry and shake confidence in human skill. Errors of diet and vitiations of the air he considered the most pregnant sources of diseases, and to the regulation of the one and purification of the other much of his success in the treatment of disease must unquestionably be ascribed. Bathing was among his favorite remedies, and the restrictions, with which he guards its employment, are almost the very same with those which Dr. Currie, in his medical reports on the effects of cold bathing, long afterwards published. Emetics he considers invaluable in the prevention of disease. In complaints seated above the liver he took blood from the arm, in those situated below it he bled from the foot, ankle, or ham, and when purging and bleeding failed to afford the necessary relief, he had recourse to sudorific and diuretic medicines. Digestion he ascribed to concoction, blood he regarded as the source of heat, and some obscure hints on the course of this fluid through the body are repeatedly thrown out.

It were irrelevant in the present place to enter more minutely into the doctrines and discoveries of Hippocrates, but it might tend to lower the pride of the present day, as well as do justice to many names which are now almost forgotten, were we to run a parallel between the state of medicine at the close of Hippocrates' career and its condition among ourselves. When we reflect that between the mysteries of theorists and the mummeries of priests, the little truth which was really known, when this great man made his appearance, was so blended with gross error, that the incongruous compound seemed almost uniform in incomprehensible absurdity, it were no mean praise to say that Hippocrates separated the dross from the metal; but when we add that he not only did this, but so augmented the sum that was really known by additional facts of his own discovery, that little, comparatively speaking, has been since done, which, in strict veracity, can be called original, our voice of praise must be raised into one bordering upon enthusiasm. Before his day medicine was a contemptible medley of silly quackery, without either principle or plan, and since his period we have rather been confirming the foundation which his industry laid down, than materially adding to the superstructure.

Thessalus and Draco, sons of Hippocrates, and Polybus, his son-in-law, followed the profession of their father, and rose to considerable eminence, more probably in consequence of their connexion than their talents. Draco was made physician to Archelaus, King of Macedonia; and Polybus succeeded his father-in-law in lecturing upon medicine. The luxurious habits, however, into which Greece fell soon after this period, had its ordinary effects upon the progress of general knowledge, and medicine, in common with other subjects of pursuit, materially suffered. The school of the Empirics, to which the Cosan Sage belonged, was succeeded by that of the Dogmatists, the country was speedily overrun with Sophists, who were perpetually disputing on the doctrine of revulsion; one party contending that the peccant humor should be removed by means applied directly to the part affected, while others argued that it should be withdrawn at some distance from it. Praxagoras was the last of the Asclepiadæ, who attained distinction. He first described the points in which arteries differ from veins, he performed some very bold operations in surgery with great success, he fancied that all the seminal principles of disease existed in the blood, he gave no medicines which were not of a vegetable nature, and he ascribed much more importance to the indications of the pulse in the management of disease than any of his predecessors.

In the fourth century before Christ Aristotle added, more especially in comparative anatomy, many important facts to the fund of medical science; but yet when we find that he gives three ventricles to the human heart, that he considers this organ as the source of the nerves, that he describes the bones of lions as destitute of marrow, and the necks of wolves as inflexible, we cannot join with any great degree of earnestness in the popular applause with which it has been fashionable to hail the preceptor of Alexander. His nephew Callisthenes wrote two works on anatomy and botany, and his pupil Theophrastus catalogued five hundred plants, describing their physical and medicinal qualities. On the death of Alexander science took up her residence in Alexandria under the auspices of Ptolemy Rater, and as the prejudices, which had hitherto prevented the cultivation of human anatomy, were gradually yielding before the light of knowledge, the study of the human body was first formally taught in Alexandria. Herophilus and Erasistratus were educated in this school, and so practised were they in the examination of the human body after death, that Tertullian, who lived nearly 500 years after Herophilus, speaks of him as a "butcher who dissected 600 men to discover nature, and who hated man in order to learn the structure of his frame." This worthy father has transgressed, we fear, the limits of truth as well as charity, when he asserts that many of the subjects which Herophilus inspected "did not die a natural death, but expired amid all the agonies to which the curiosity of the anatomist was pleased to inflict upon them." Something of the nervous system was for the first time revealed by this man, yet he falls into the universal error of his predecessors, in confounding tendons and ligaments with nerves, and from his predilection for soothing remedies Galen calls him an empiric. Erasistratus, his cotemporary, by discovering the lacteals, added materially to the rising reputation of the Alexandrian school, and yet it is a fact so singular as scarcely to be accounted for that, adopting the fancy of the Egyptian dogmatists, he believed the arteries to be filled with air. Erasistratus lies under the same imputation which Tertullian fastened upon Herophilus, and it is more than singular that such a man as Celsus could be betrayed into the belief that the charge was well founded. He says that both Herophilus and Erasistratus "dissected living criminals condemned to death, and dragged them from their prisons for that purpose."

About this period the medical art was divided into surgery, pharmacy, and medicine—a triform character which it has since worn, and now instead of practising generally, as had been previously the case, those who treated internal disease affected a superiority over such as performed manual operations, and the surgeon and physician combined in making the pharmaceutist inferior to both. The evil, which these absurd distinctions have since done to the interests of our profession, it were probably difficult to estimate; but this we apprehend can be fairly established, that any good, which it may have produced, falls far short of counterbalancing the ills to which it has given rise. Shortly after this Utopian distinction was established, the profession became distracted into opposing sects, and a spirit of envy gradually crept into it which marred the harmony that had hitherto prevailed; many confined themselves not only to the performance of a single operation, but to the preparation of a single remedy which, like some of our patent drugs in the present day, was christened in the name of its inventor and puffed into notoriety as infallible.

After the seat of empire was transferred to Europe, Rome became the nursery of knowledge, and many of the most distinguished Grecian and Egyptian schools, following the seat of power, took up their abode in Italy. At first, however, the Romans betrayed an almost invincible aversion against medicine, nor was this feeling of dislike a vulgar prejudice, which was confined to the lower orders of the poor, for no less distinguished a man than Cato the Censor dispensed with professional assistance during any sickness in his family, preferring to treat them according to the directions of a work in his possession which contained the necessary forms of prayers and incantations. It is curious to perceive how a man, so justly distinguished for superior talents and acquirements, should fancy that dislocations could be reduced or fractures healed by such unintelligible jargon as "huat, hanat, huat, ista, pista, sista, domiabo, damnaustra," and "huat, haut, haut, ista, sistar, sis, ardannabon, damnaustra." Fear, however, at length accomplished what reason could not achieve, and the violence of an epidemic, which took place nearly four centuries after the foundation of their city, and which baffled all their charms and incantations, drove them for advice to the temple of Epidaurus. Æsculapius, it would appear, was so propitious to the supplicants that the plague subsided shortly after their visit to the oracle, and not only was a temple erected in gratitude to that deity, but various other new gods, whose names had scarcely been previously known, had distinguished honors paid to them. Hence a votive tablet was erected to the goddess Febris, and the goddesses Assipages, who presided over the growth of bones, and Carna, to whose care the viscera fell, were canonized. One of the first men of note, who settled in Rome at this period, was Asclepiades, who was a Materialist and who treated the body as a mere machine. Fevers, inflammations and similar disorders of excitement he ascribed to obstruction, while dropsy, languor and syncope were referred to relaxation of the pores. Had this mountebank been content with forming theories which he could not establish, the peculiarities of his system might not have excited much attention; but he attacked the doctrines and practice of Hippocrates with such an unsparing hand, that an artificial importance was thus attached to what might otherwise have lain long neglected, and as wine was his sheet-anchor in every disease, it has been imagined by some that to the exhilarating effects of this soothing cordial he was mainly indebted for his popularity. After his death Themison his pupil established the methodic sect, which Saranus, Thessalus and Cœlius Aurelianus afterwards raised to very considerable distinction.

Although medicine had now attained a firm and reputable rank among the sciences, which were taught in Rome, it is somewhat singular that not one of those, whom we have mentioned as having contributed to raise it to this distinction, was of Roman origin; the majority having been Greeks and some Egyptians. Aurelius Cornelius Celsus is the first Roman citizen, whose medical works have descended to us, yet some have found reason to suspect that he himself never practised physic. However this may be his writings are much superior to all those which preceded them, whether we consider the number and importance of the facts which they contain, the perspicuity and good taste with which these facts are detailed, or the caution which has been observed in mixing up any thing very conjectural with the practical subjects which engrossed his attention. Equal to Cicero in the classicality of his style, and to Hippocrates in the practicality of his matter, he excelled all preceding writers in the unrestrained candor with which he acknowledges truth in whatever sect he discovered it, and in the diligence with which he selects from their various systems whatever appeared to him judicious and valuable. Like his great predecessor Hippocrates he was strictly an eclectic, who neither acknowledged sects nor schisms any farther than the doctrines which they taught were consonant with reason and verified in practice. His parentage is utterly unknown, but whether it were patrician or plebeian is of little importance to posterity, and certainly of none whatever either to the perfection of his character, or the perpetuity of his fame.

About the middle of the first century, and nearly if not cotemporary with Celsus, appeared the *incomparable* Aretæus as he was for a long time designated, from the value and elegance of his writings. First a pneumatist he afterwards attached himself to the eclectic school, and by advocating the necessity of anatomical knowledge to the successful treatment of disease he eagerly contributed to place the practice of medicine on that scientific footing, which regulates our curative measures according to the structure and functions of the diseased organ. He may justly be esteemed the father of pathology, as little if any attention was paid prior to his day to the structural changes, which morbid actions occasion. He always bled on the side opposite to that affected, he introduced cantharides into medicine, and although he prescribed few remedies, yet when obvious indications of treatment existed, he combated the danger with an uncompromising firmness, which many considered bordering on rashness. This censure we believe was totally groundless, for the pathological principles, by which Aretæus regulated both the character and action of his remedies, were much more precise and less calculated to deceive than those theoretical dogmata, which so generally occupied the attention of the schools.

In travelling along the historical line of our profession, the next brilliant ornament which adorns its annals is Claudius Galen. This celebrated Grecian father was a native of Pergamus, who, after finishing his education in Alexandria, settled in Rome in his 34th year, and shortly afterwards commenced a course of lectures on anatomy, which displayed so much talent, and excited so much attention, that the Roman physicians became jealous of his rising fame, and, by a series of harrassing persecutions, compelled him to purchase peace by withdrawing entirely from the city. Scarcely, however, had he spent a year in exile when he was recalled by Marcus Aurelius, and, as soon as Commodus came to the throne, he was appointed physician to the young emperor. His writings made their first appearance in the world anonymously, whether from modesty or cunning is not certainly known; but, as Galen was remarkable for his high opinion of himself, and as part of the persecution which he endured arose from an indiscreet manifestation of this consciousness, it is not likely that diffidence was much concerned in this arrangement. One, who could declare that the glory of pointing out the true path to medical science was the property of Hippocrates, while that of conquering the obstacles which rendered its approach arduous was reserved for him, was not very likely to withhold his name from a title-page through fear of being overwhelmed with praise. On medical subjects alone he penned between 4 and 500 treatises; and, unlike many writers, who plead the voluminousness of their works as an apology for their incorrectness, every subject upon which he wrote is carefully considered, and treated with as much care as though no other shared its claims upon his attention. His indiscriminating reverence for Hippocrates seduced him into a credulous admission of all that sage had taught. Whatever the Coan chief asserted he found reasons to defend, and no strength of argument was found sufficient to recommend whatever had encountered his opposition. Hence may we, perhaps, explain some of the gross anatomical and practical blunders with which his works are mutilated; such as his tracing the origin of the veins to the liver, and of the arteries to the heart; and to his overweening love of theory may many other deficiencies be referred. In this respect, these two great men most widely differed. Conjecture was never hazarded by the one, unless it were strongly supported by appearances, while the other was seldom proof against theory, when its aptitude promised a ready explanation of what seemed mysterious or abstruse. When young, he strongly inclined to atheism, but his anatomical studies had the effect of awaking him to a belief in the Supreme Being, and his writings are strewed with the most pious reflections upon the beneficence and wisdom of his government.

It is generally believed that, for the space of 1200 years from the days of Galen, medicine, like every other science, remained stationary; yet, although the labors of Aribasius, Numesius,

Œtius, Paulus Ægineta, and a few other writers, whose names have survived the general ruin, are too few and unimportant to falsify an observation which is generally accurate, still some injurious errors were expunged, and a few important additions had been made. Several surgical operations were performed which had not been previously attempted, some remedies were discovered which had been previously unknown, and, upon comparing the writings of Œtius with those of Galen, it is quite certain that the catalogue of human maladies recorded by the former is one-third larger than that which the latter had described. The obstetrical branch of the profession was more especially a gainer during this dark and unenlightened period. Indeed, until Paulus Ægineta contrived to gain the favor of the Arabian ladies in this department, midwifery seems to have beeen almost entirely neglected. When the dimensions of the pelvis rendered parturition impracticable, embryotomy was proposed by him; but the previous death of the child he considered a *sine quâ non* to its performance.

As the downfal of the Roman Empire, by the incursion of the northern tribes, extinguished the torch of science in the West, the capture of Alexandria by the Caliph Omar, and the destruction of the Alexandrian Library by that ignorant enthusiast, arrested the progress of knowledge in Egypt, and transferred into Arabia most of the distinguished philosophers of that day. By the exertions of John Philopomus, and some other friends to learning, some important manuscripts were rescued out of the hands of the Mahommedan. These papers the Syrian Christians carefully translated into the language of their country, and, towards the close of the eighth century, medicine had so far revived from the injury which it had sustained, that the Caliph Almanzor established a college at Bagdad, to which his famous successor, Haroun al Raschid, added many public schools and hospitals. Mesue the elder, Serapion, Rhazes, Hally-Abbas, Avicenna, Albucasis, and Avenzoar, are among the most celebrated men to which these seminaries gave birth. Ali Asbaia enumerates no fewer than 220 treatises of which Rhazes was the author, and the Arabian historians describe him as being as skilled in astronomy, chemistry, and every other science then cultivated, as he was in medicine. From their belief that the soul remained in the body for a considerable time after death, human anatomy was strictly prohibited by the Arabians. No improvement, therefore, of any consequence seems to have been made in this department, but both surgery and physic advanced under their exertions. Spina bifida, small-pox, and measles were accurately described, for the first time, by Rhazes, trichiasis was cured by him, (as we do) by cutting out a slip of the eyelid, fistulous ulcers and indolent sores were treated by compression, and chemical medicines were first introduced by the Arabians into the management of disease. Their knowledge of pharmacy generally was very considerable; one of the directors of their college at Jandisabour gave the first dispensatory to the world, and the municipal authorities not only watched over the qualities, but regulated the prices of their drugs. The Hawi of Rhazes and the Canon of Avicenna fully rivalled the writings of the Grecian fathers, both in the extent of their circulation and in the influence of their doctrines. For five centuries, Avicenna was as faithfully followed as though his writings had been dictated by the spirit of infallibility, and so great was the reverence which his talents and acquirements inspired, that the gross immoralities of his private character, in place of detracting from his fame, seemed to operate as a foil to his public virtues. Avenzoar, who flourished not long after his decease, and whose professional character was little, if at all, inferior, held him in the very highest estimation, calling him the "treasury of universal knowledge," and his works were translated, abridged, and commented on, for nearly six hundred years after his death.

Soon after this period, the arts and sciences declined among the Saracens, and, by the establishment of an intolerant government, the Turkish power speedily extinguished them. Salernum, a small town within the territories of Naples, now became the refuge of knowledge. From a very early period, there had existed in this city a monastery of Benedictine Fathers, and, although medicine was at first generally practised by the priests for the sake of gain, and in total ignorance of even its most fundamental principles, about the beginning of the 10th century, these monks began to apply themselves more regularly to the study of medicine, and, taking the works of Galen for their guide, they gradually rose in reputation, and materially contributed to its improvement. Towards the close of the 12th century, Benjamin of Tudela visited Salernum, while travelling in quest of his countrymen in the East; and so highly had it risen in character above every other seminary, that this Jewish Rabbi describes it as the best school of medicine among the children of Edom. Frederick the 2d conferred upon this school, in the year 1225, the power of conferring degrees on its pupils, both in medicine and surgery; it was placed under the protection of St. Matthew, and was styled "Civitas Hippocratis." The examinations were conducted with the greatest strictness; candidates for the degree of Dr. in medicine were required to produce seven years' tickets from competent professors, and they were obliged to study as text-books the Therapeutics of Galen, the first Canon of Avicenna, and the Aphorisms of Hippocrates. Before admission could be obtained to a surgeon's diploma, twelve months' attendance on anatomical instruction was considered necessary; and, on being admitted, the candidate was sworn to refuse remuneration for attendance upon the poor, and to enter into no lucrative compact with druggists or apothecaries. These preliminaries gone through, a book was placed in his hand, a ring on his finger, a crown of laurel upon his brow, and he was hailed with the kiss of peace.

The commencement of the 13th century was distinguished by the appearance of two men, whose exertions in behalf of knowledge, at a period of such general darkness, obtained for them among their cotemporaries the character of magicians. Roger Bacon in England, and Albertus Magnus in France, gave an impulse to the progress of learning which it had not before received since the decline of the Arabian schools. The prelates and the monks, however, rose up in arms against the zealous exertions of these enlightened men. Albertus was denounced as an arch magician, and, although Bacon was himself a Franciscan Monk, the General of his own order persecuted him with such unrelenting severity, that he kept him imprisoned for upwards of ten years. The anxiety which existed at this time to discover the philosopher's stone, contributed to direct the attention of these men more immediately to chemistry. The *aurum potabile* was, in Bacon's estimation, a most valuable remedy, and he extols, in no very measured language, the restorative virtues of *vipers' flesh.* Medicine had now become a general branch of education in the principal schools of Europe, but the universities of Montpellier, Paris, Bologna, Padua, Milan, Pavia, and Piacenza, were most esteemed in this department.

England had not, as yet, done any thing to advance the interests of medicine, if we except the eight general treatises of Friar Bacon, which were principally collated out of the writings of the ancients. No encouragement was held out by the schools in England at this period for the cultivation of medicine. The treatment of disease was exclusively the province of the monks, and their influence over the people's mind gave them such an advantage over lay practitioners, that, for a long time, these last could not undertake to rival them with any prospect of success. Gilbertus Anglicanus may be fairly considered as the first medical writer of any note to which our country gave birth. His principal work was a compendium of medicine; but he did more essential service to the profession, by exposing the ignorance of the monks in the knowledge and treatment of disease, than by his writings, which contained very little that might not be found in the works of the Arabians. Gilbert is supposed to have flourished in the beginning of the 14th century, and, about the year 1320, appeared John of Gaddesden, the author of the *Rosa Anglicana*, which Chaucer's muse has so laboured to extol. He pretended to cure all diseases, and, where ordinary remedies proved fruitless, he was not backward in having recourse to secret charms and unknown nostrums. He was appointed physician to Edward II. and, as the court physician had ever previously been a foreigner, the distinction conferred by such appointment must have been considerable.

What John did for medicine, Guy de Chauliac achieved for surgery. This man was professor at Montpellier, and, after practising for many years at Leyden, he ultimately settled at Avignon, where he became physician to Clement VI. In his *Magna Chirurgia*, which has been translated into most of the European languages, he gives us a list of the principal writers upon surgery before him, and annexes to each a short critique on the doctrines and practice recommended. He minutely details the several modes of treating hernia by the knife, the caustic and the cautery, and he was the first to observe that the Cæsarean operation may be practised after the mother's death to preserve the child. Cotemporary with Guy was John Ardern, whose treatise on *fistula in ano* was translated as late as 1588. He is generally considered to have been an Englishman, but there are reasons for suspecting his birth-place to have been further north. In cautious prudence and contracted selfishness he was obviously Scotch; for he always bargained with his patients before entering upon their treatment, as to the amount of remuneration with which his labors would be repaid, and, not satisfied with acting so avariciously himself, he advises medical men to stipulate for as much as they can get, and to obtain security for the payment of the sum agreed upon as soon as the cure is accomplished!

About the commencement of the 15th century Jacobus Sylvius flourished as a lecturer in Paris, and immortalized his name by important discoveries in anatomy. His reverence for Galen was so great as to interfere with his private friendships; and, although Vesalius had been his own pupil, he wrote several books against him, because he had the audacity to criticize his favorite father. Surgery gained much more from the labors of this century than did the science of internal disease. Even lithotomy, which had been considered an impracticable operation, and to refrain from which Hippocrates regarded so essential, that *ne vero calculo laborantes secabo* was part of the oath which he administered to his pupils, was successfully performed. Germain Colot was the first regular surgeon to break through this patriarchal precept; for, strange to say, an operation so difficult and so dangerous, as to call forth such marked prohibition, had been practised for a long time previously by a set of itinerant quacks, whose ignorance of surgery could have held out to them but little chance of success. Louis XIth., upon the urgent entreaties of his court physicians, gave Colot permission to try his knife upon a condemned criminal, who happened to be labouring under calculus; and this man was so rapidly restored to health, being convalescent in the short space of fifteen days, that Colot's talents were rewarded with a pension, and lithotomy became in the hands of educated surgeons a regular part of surgical practice.

Two diseases made their appearance in England about this period, one of which has continued to ravage its inhabitants ever since, while the other would appear to have become extinct since the year 1575. The "Sudor Anglicanus" was in many respects a peculiar disease, exhibiting in its symptoms an anomalous compound of ague, plague, and putrid fever. It generally proved fatal in the course of 24 hours, and its partialities were very extraordinary. In its first six visits to this country in 1485, 1506, 1518, 1528, 1529, and 1551, it confined itself almost exclusively to the English, leaving foreigners, and even the Irish and Scotch, unmolested; while during its last attack in 1575, it carried off in 36 days no fewer than 510 persons, all of whom were males. To what cause its first appearance was attributable is unknown, and why it should have never since returned is equally inexplicable. Syphilis—the second of the diseases above alluded to —was almost equally destructive with the sweating sickness on its first arrival into Europe. Such was the consternation it excited in Edinburgh, that the inhabitants, fancying it to be a contagious plague capable of spreading from person to person without immediate contact, banished all who became affected with it to the Island of Inch-Keith, where they were doomed to perform quarantine until restored to health. Marcellus Cumanus seems to have been the first who formally wrote upon this disease. His work was followed by one from Leonicenus, a Professor of Ferrara, but Casper Torella is more minute than either in describing the character of the ulcers, and the most suitable mode of treatment. His plan of cure consisted exclusively of purgatives, bleeding, baths, and diluents. Mercurial frictions he reprobates as highly deleterious, and he gives the formulæ of two mercurial ointments which, he says, had in the hands of empirics occasioned death to multitudes. Like the modern anti-mercurialists among ourselves, he treated syphilis as one of a purely inflammatory character, which mercury was more calculated to aggravate than cure. Jacobus Cataneus strongly advocates the cause of mercury, advising its employment until the gums swell, and he states it as his conviction, that the syphilitic virus may remain in the system for 30 years before it betrays its presence by external signs. Gonzalo Fernandez, becoming affected with this disease at the seige of Naples in 1794, and finding no practitioners in Italy acquainted with its management, embarked for the West Indies, where he understood syphilis was well known, and returned with Guaiacum as the herb which had accomplished his cure. If we are to believe that the venereal disease came to us from the West Indies, it is a fact somewhat curious in its coincidence, that one of the most popular, and certainly not least inactive counter-agents to this poison, should have been derived from the same quarter. The station, however, which mercury had assumed in the cure of syphilis, was not altered by the arrival of this new remedy; for both John de Vigo and Berengarius were in 1518 so highly successful in the use of mercurial frictions, that they made very large fortunes by pursuing this branch of practice alone.

At this period the preliminary ordeal, through which candidates for the medical profession were required to pass, was extremely loose and insufficient. The Bishops were vested with the important prerogative of admitting to the practice of medicine, within their respective dioceses, such as applied for examination; and as the clergy had at this time been almost entirely banished from the actual pale of the faculty, few seeking their advice in preference to that of regularly educated men, it is not difficult to conceive that it neither required much study nor talent to pass with eclat through such examinations, as these reverend prelates would find it convenient to undertake. Thomas Linacre, a native of Dover, who had enjoyed peculiar opportunities, while studying upon the Continent, of witnessing the consequences of such a wretched censorship, set about redressing this grievance, and from his influence with Henry VIIIth. through the interference of Cardinal Wolsey, our present College of Physicians was established in 1518, and was invested with the exclusive right of examining the pretensions of all, who desired to enter the profession. Linacre was made president of the College he had thus established, and upon his death, seven years afterwards, he bequeathed to it his house in Knight-Rider Street, where all their meetings had hitherto been convened. The influence, which this establishment exerted upon the character of the profession, as well as upon the progress of medicine, was as great as it was sudden. Much fewer candidates applied for examination, because the test of their qualifications had been made much more rigid; and those, who were considered deserving of admission, generally confirmed the choice of the College by the rank and respectability of their future character. How far the same effects can be traced to the same source in the present day, it were deviating from our purpose to consider; but, however we may regard the College of Physicians, as it is now constituted and conducted, there can be no doubt but that the exigences of the times which called it forth demanded the establishment of some such institution.

Three years after Linacre's decease, the celebrated Paracelsus began to lecture at Basil; and, although possessed of the *elixir vitæ*, which was said to have had the power of prolonging life to any period, like all other empirical impostors he stultified his pretensions in his own case, for he died of fever at the early age of 48. Fracastorius published a poem upon syphilis in 1530 at Verona, which was so classically written as to have been compared by tolerably competent judges to the Georgics of Virgil. Ambrose Paré published fifteen years afterwards his first work on the treatment of gun-shot wounds, in which he describes for the first time, at least with accuracy, the operation of restraining hæmorrhage by passing a ligature round the bleeding vessel. In 1583 Botallus' work on bloodletting made its appearance, and excited very general notice. At the commencement of the 16th century Peter Brissot, a physician of Poitou, met with violent opposition for having advocated bleeding from the same side with the inflamed part in the treatment of pleurisy; and Denys, physician to the then King of Portugal, not only denounced such practice as dangerous, but as destructive to the body as Luther's doctrines were to the soul. Many there were, however, who neither sanctioned bleeding on the same side with the disease, nor on that opposite, in the great majority of inflammations; and it was to combat the objections of this party that Botallus' Essay, "De Curatione per Sanguinis Missionem," was penned. He advises bleeding in diarrhœa, dysentery, fever and plague; in pregnancy, where it was all but prohibited, he recommends it; he has the priority of Blackall in urging the use of the lancet in dropsy, and Dr. M'Intosh may read with some advantage his remarks on its applicability to cases of quartan ague. Like most *monomedici*, however, he pushed his advocacy of an invaluable remedy to an extreme. He records cases in which he repeated the operation beyond the 18th time, and in the days of Le Sage venesection, by being abused, had become so obnoxious to censure, that the French satirist makes Dr. Sangrado, in his History of Gil Blas, the ridiculous representative of the Botallus party.

While medicine and surgery were thus rapidly advancing towards a more perfect state, anatomy was not neglected. It was in the 16th century that Servetus in his work *De Christianismi Restitutione*, laid the foundation-stone of that superstructure, which Harvey afterwards raised on the circulation of the blood. Some, from an over zeal for our illustrious countryman, have endeavoured to deny that any thing was known upon this subject before his day; but such advocacy is injudicious, and tends only to lower a reputation which is sufficiently unassailable on higher grounds. That Servetus knew something of the pulmonary circulation there cannot be a doubt in the minds of those, who have perused the work to which allusion has been made, and we are not quite satisfied that the chemical alterations, which the venous blood experiences in the lungs, were totally unknown to him. During this century the Pancreas was first so called and described by Guinterius; the olfactory nerves are traced by Dryander; the great work of Vesalius, "De Humani Corporis Fabrica," was published; Realdus Columbus discovered the tunica innominata of the eye; Ingrassias the stapes of the ear, and the names of Fallopius, Eustachius, Varolius, Cæsalpinus, and Fabricius, require only to be enumerated, to show the progress which anatomy was making at this period.

Early in the 17th century the writings of Van Helmont began to attract the notice of the profession. Forgetting the reverence which his predecessors entertained for the doctrines of Galen, he openly attacked the writings of this Father, and after a considerable contest succeeded in shewing that his hypothesis of the four qualities, with their four degrees, upon the existence of which he had erected his entire system, was perfectly gratuitous and fanciful. The practice, to which this system led, he also severely criticised, and as chemistry was his favorite science, like his predecessor Paracelsus, he referred every fact to some chemical law, and solved every difficulty by some chemical principle. For many years the chemical school, which was thus established, possessed considerable popularity; but, although afterwards much improved upon by Francis Sylvius, the works of Van Helmont are now only regarded as speculative curiosities.

About the period of his death Glisson, the anatomist, was in the habit of meeting some literary friends in London for the purpose of discussing subjects connected with philosophy; but their numbers so increased, and the mutual advantages, which were derived from these primitive *conversaziones*, encouraged Glisson to apply to Government for a royal charter, which was granted to them after the Restoration. The *Royal Society* thus founded by a few enterprizing individuals, whose first object in associating was principally entertainment, soon attracted the notice of all Europe, and until the honors, which it was privileged to confer, began to be indiscriminately lavished upon many, whose only claim was enormous wealth, patrician rank, or political influence, to be a fellow or even member of this body, was regarded one of the highest honors, which could reward industry or distinguish merit. About this time the forceps was reaping a golden harvest to the Chamberlains in the practice of midwifery, and from Julian Clement's success in the accouchement of the Duchess de la Valière, it became fashionable to substitute male for female practitioners in such cases. Clement simplified the operation of turning in false presentations, he proposed rupturing the membranes during the first stage of labor in cases of hemorrhage, and he abolished many very injurious customs, which had been adopted in the treatment of the puerperal state, from the persuasion that lying-in women should be considered in all respects as labouring under the influence of ordinary disease. Deventer endeavoured to purchase Chamberlain's secret, but finding its price too extravagant for his means, he took up the cases in which Chamberlain had proved unfortunate, and thus strove to compensate his loss by shewing the injurious effects of instrumental midwifery. In difficult labors, where the pelvis seemed confined, Daventer used to enlarge the inferior outlet, by pushing back the os coccygis, and when an arm presented he turned the child and delivered by the feet, in place of adopting the advice of preceding writers, which recommended amputation of the limb.

In 1622 Asellius claimed the discovery of the lacteals, although these vessels had certainly been described by previous anatomists; six years afterwards, Harvey laboriously demonstrated the circulation of the blood; and in 1651, Rudbeck contested with Bartholine and Joliffe the merit of first describing the lymphatic system. A century before, the thoracic duct had been seen by Eustachius, but Pecquet, because he demonstrated how it was formed by the conjunction of innumerable branches arising from the intestines, and how it terminated, not in the liver as had been imagined, but in the veins near the heart, has been very generally regarded as the discoverer. In 1656 Wharton published his elaborate treatise on the glands; the year after Highmore laid claim to the discovery of the sinus which now bears his name, although already described by Casserius; and the parotid duct has been *christened* with the name of Steno, because he described it in 1662, although Blasius had previously pointed it out to him. It has been often said that posterity are generally just in their rewards of merit, but if he, who is a believer in this proverb, trace attentively the history of discoveries, few popular dogmata will be found more baseless. In the course of this article we have occasionally noticed some instances corroborative of this opinion, and it might be doing a service to some of our *original writers* in the present day, were we at a future opportunity to enter more largely into detail.

Malpighi by his microscope and Ruysch by his injections threw a new light upon many parts, which had been but imperfectly understood before, and brought many others into view which had been hitherto overlooked. The opinions of these two distinguished men on the ultimate form of glandular structure gave rise to a controversy, which burned with vigor for a considerable time, and which proved the means of eliciting much valuable light upon minute anatomy. Ruysch died in his 93d year, and whether we contemplate the value of his discoveries, the splendor of his preparations, or the number of his works, we have no anatomist of even more modern days, whose services to that branch of the profession stand so pre-eminently unrivalled. Meibomius, Borelli, Swammerdam, De Graaf and Bonetus were working assiduously at the same time with Ruysch, and although less successful in their labors their discoveries were neither few, nor unimportant. Our countryman Cowper, over-anxious to descend along the stream of time in company with such names, involved himself in an uncreditable controversy with Bidloo, for having meanly pirated a number of his plates.

With so many vestiges of rapid progress, as we have now hastily traced, and the principal only have been specified, it may appear strange that Sydenham should have thought so contemptibly of the state of medicine in his time, that when consulted as to the best authors to be perused, he advised the inquirer to "read Don Quixote"! But, while the words of this reply indicate the state of feeling which gave rise to it, we cannot deny but that many of Sydenham's cotemporaries, not to mention his predecessors, were as romantic and as fanciful as was this chivalrous knight. Borelli was at this very moment, in his chair at Pisa, labouring to explain all the functions of life upon purely mathematical principles, and Borri, who was one of the most impudent impostors, which ever infested society, was travelling in state through the principal Courts of Europe, having for his patients potentates and kings! No less a period than two thousand years had now elapsed, since the days of Hippocrates, and theory and speculation were still as liberally indulged in, as though that Grecian sage had never inculcated the importance of cool and deliberate observation. One party fancied that they could explain all the phenomena of disease by mechanical, others upon chemical principles, and believing that the human body was subject to all the laws of dead matter, they had almost wholly excluded from their calculations the principle and influence of life. Sydenham might, therefore, justly say that there was neither certainty nor satisfaction in the medical knowledge of his day, and that the history of the Knight of La Mancha might be consulted, if not with almost as much profit, at least with quite as much entertainment. He substituted patient observation for hasty inference, sober induction for intemperate generalization, and the careful study of natural appearances for the faithless pencilling of a sanguine fancy. His histories of small-pox, gout and measles are not to be excelled by any descriptions of the present day, and his view of fever and of other acute diseases is much more agreeable to nature and more conducive to practice, than many which within the last few years it has been our lot to criticise.

About 20 years after the death of Sydenham, Herman Boerhaave succeeded Drelincourt in the chair of medicine at the University of Leyden; and, although in the course of a few years he became the head of medical instruction in every department in that city, as a physician he was consulted by the inhabitants of every country, as a chemist he was second only to Stahl, and his lectures upon botany attracted much attention. His doctrines rapidly spread over Europe, he was elected a Fellow of the Royal Society, and a Foreign Associate of the Royal Academy of Sciences at Paris. Cotemporary with Boerhaave was our illustrious countryman Mead, who attained an almost equal degree of celebrity by the number of his works and the distinguished superiority of his talents. Being applied to by the Lords of the regency, when the plague was raging with violence at Marseilles in 1719, to inquire into the best means of preventing its introduction into England, he published a treatise on this disease in the following year, which was bought up with such avidity, that no fewer than seven editions were purchased in twelve months. His work on poisons is a standard production at even the present day, although many of his views as to their operation are too mechanical; and his treatise on sol-lunar influence should be consulted by those, who dispute the agency of the moon upon the propagation and progress of disease.

The beginning of the 18th century was distinguished by the birth of Haller. This most illustrious philosopher commenced his career of fame when only ten years old, by writing a satire in Latin verse against his tutor; and during the remaining 58 years of his active life, his contributions to almost every branch of medical science it were a task even to enumerate. Before his day physiology was scarcely known as a distinct subject for investigation, but by carefully arranging the scattered facts pertaining to it which had been previously ascertained, and by instituting experiments upon points which had either been before unexamined or unsettled, the science of function began to assume in his hands a specific form.

About ten years before Haller's death medicine began to set up her schools in the New World, and to rival Europe in the celebrity of her sons. Lectures on anatomy and surgery were first delivered by Dr. Shippen in 1764, in Philadelphia; in 1765 practice of physic was taught by Dr. Morgan; in 1768 Dr. Kuhn added botany and materia medica to the other subjects for lecture; and 1769 strengthed this rising seminary with the distinguished talents of Benjamin Rush. In 1791 the medical school of Philadelphia was incorporated with the University of Pensylvania, Dr. Rush was appointed Professor of the Institutes and Practice of Medicine, and from that time forward our transatlantic brethren have not only been gradually rising in reputation, but they have been keeping tolerably equal pace with the parent country both in the number and value of their writings.

With this running outline of Mr. Moir's and Dr. Hamilton's works we must drop our pen, and as we have refrained from interrupting the continuity of our historical sketch by any passing observations upon the historians themselves, we cannot dismiss the subject without a word or two upon the style and conduct of their respective performances. In medical as well as in general history, fidelity of description, beauty of expression, a discriminating selection of subject, and an impartial review of doubtful points are the essential requisites of an able and faithful historian, and we are happy to say that in several of these respects Dr. Hamilton's pen is highly qualified. The language he employs is beautiful and occasionally eloquent, his impartiality is obvious throughout, he is often very fortunate in the selection of his subjects, and when the sterility of the soil, through which he is occasionally compelled to travel, would give an aridity and insignificance to his narrative, imagination not unfrequently gathers flowers from more favoured districts, and strewing them beneath the traveller's feet thus conceals the nakedness of the land.

Many points, it must be confessed, in the history of our profession are any thing but inviting to the historian's pen. Theories very vaguely conceived and still more imperfectly supported, facts obscurely ascertained and fancies crude as they are extravagant, are too often the only materials which can be discovered, during lengthened periods in the progress of our art; and the very nature of the science, abstractedly considered, throws an uninviting sameness of character over even the brightest days in the sunshine of its history. Considerable judgment and more tact are, therefore, necessary to impart sufficient interest to a history of medicine, which is written for general readers; and if we say that the efforts of Dr. Hamilton have proved somewhat unsuccessful, the severity of the criticism will be considerably mellowed by considering the difficulties which the author has had to encounter to avoid it. Had the Dr. passed more lightly over the early periods of his subject, when there was nothing but darkness, confusion and uncertainty, and dwelt more largely upon men and doctrines nearer to our own day; had he taken up more formally the different sects which at various times arose, and while tracing their rise and fall, had he used them as the bond of transition by which his narrative ran from century to century, the professional reader had been more instructed, and the general reader would not have been less entertained. Half a dozen writers at the close of the 18th century would have furnished a richer subject for observation than twice as many cycles in the centre of the dark ages; and yet the names and achievements of Haller, Rush, Franklin, Heberden, Monro, Black, Cullen, Brown, Gregory, Hunter, Baillie, and Bell are passed over in almost as many pages, while Bovius, Paracelsus, Asclepiades, Themison, Thessalus, Borri and such quacks and mountebanks occupy as much space and receive as much attention.

As Mr. Moir confined himself to an historical sketch of ancient medicine, he was necessarily limited by his subject; but Dr. Hamilton's pen was not so restricted, and if pleasure and improvement be the objects contemplated by the historian, either would be more surely attained by making the interest of the period, or the value of the writer, the measure of the limits to which his notice should extend. As a manual of the ancient history of medicine Mr. Moir's is a concise, correct and judicious performance, which is not less elegant than Dr. Hamilton's, although it is more condensed. It may be read with more ease than Dr. Millar's learned and laborious history, while it may be consulted with equal profit; and in the two volumes of Dr. Hamilton the reader will find as much of the substance of Sprengel's works, as may make him sufficiently well acquainted with the different eras in the annals of his profession, without toiling over the pages of that voluminous chronicler.

